# Characterisation of mobile colistin resistance genes (*mcr-3* and *mcr-5*) in river and storm water in regions of the Western Cape of South Africa

**DOI:** 10.1186/s13756-021-00963-2

**Published:** 2021-06-29

**Authors:** Yolandi Snyman, Andrew C. Whitelaw, Jo M. Barnes, Motlatji R. B. Maloba, Mae Newton-Foot

**Affiliations:** 1 Division of Medical Microbiology, Department of Pathology, Stellenbosch University, Cape Town, South Africa; 2 National Health Laboratory Service, Tygerberg Hospital, Cape Town, South Africa; 3 Division of Community Health, Department Epidemiology, Stellenbosch University, Cape Town, South Africa; 4 Department of Medical Microbiology, University of the Free State, Bloemfontein, South Africa; 5 National Health Laboratory Service, Universitas Hospital, Bloemfontein, South Africa

**Keywords:** *mcr-3.33*, *mcr-3.34*, *mcr-3.35*, *mcr-3.36*, *mcr-3.37*, *mcr-5.1*, Colistin resistance, South Africa, River water, Storm water

## Abstract

**Background:**

Colistin is regarded as a last-resort antimicrobial against multi-drug resistant Gram-negative bacteria (GNB), therefore the dissemination of colistin resistance in the environment is of great concern. Horizontal transfer of mobile colistin resistance (*mcr*) genes to potential pathogens poses a serious problem. This study aimed to describe the presence of colistin resistant GNB and *mcr* genes in river and storm water in regions of the Western Cape.

**Methods:**

Water samples were collected from three rivers during May 2019 and January 2020 and two storm water samples were collected in November 2019. Colistin resistant GNB were cultured on MacConkey agar containing colistin and identified by MALDI-TOF. Colistin resistance was confirmed using broth microdilution (BMD). *mcr-1-5* genes were detected by PCR performed directly on the water samples and on the colistin resistant isolates. *mcr* functionality was assessed by BMD after cloning the *mcr* genes into pET-48b(+) and expression in SHuffle T7 *E. coli*.

**Results:**

*mcr-5.1* and various *mcr-3* gene variants were detected in the Plankenburg-, Eerste- and Berg rivers and in storm water from Muizenberg, and only *mcr-5.1* was detected in storm water from Fish Hoek. Colistin resistant GNB were isolated from all of the water sources. *Aeromonas* spp. were the most common colistin resistant organisms detected in the water sources; 25% (6/24) of colistin resistant *Aeromonas* spp. isolated from the Berg river contained novel *mcr-3* variants; *mcr-3.33* (n = 1), *mcr-3.34* (n = 1) *mcr-3.35* (n = 1) *mcr-3.36* (n = 2) and *mcr-3.37* (n = 1), which were confirmed to confer colistin resistance.

**Conclusions:**

The *mcr-5.1* and *mcr-3* colistin resistance gene variants were present in widely dispersed water sources in regions of the Western Cape. The *mcr* genes were only detected in water sampled downstream of and alongside communities, suggesting that their presence is driven by human influence/contamination. This is the first documentation of *mcr-3* and *mcr-5* gene variants in any setting in South Africa. Spill-over of these genes to communities could result in horizontal gene transfer to pathogenic bacteria, exacerbating the challenge of controlling multidrug resistant GNB infections.

**Supplementary Information:**

The online version contains supplementary material available at 10.1186/s13756-021-00963-2.

## Introduction

The emergence and spread of mobile colistin resistance (*mcr*) genes threaten the efficacy of colistin, a last resort antibiotic for treating infections caused by multidrug-resistant Gram-negative bacteria (GNB). Colistin resistance can also arise from chromosomal mutations, including those in genes encoding the two-component systems PmrAB and PhoPQ in Enterobacterales. However, in contrast to the *mcr* genes, these are not horizontally transferable.

Ten *mcr* genes, *mcr-1* (30 variants), *mcr-2* (7 variants), *mcr-3* (40 variants), *mcr-4* (6 variants), *mcr-5* (4 variants), *mcr-6*, *mcr-7*, *mcr-8* (3 variants), *mcr-9* (3 variants) and *mcr-10* [1–10], have been described in various GNB, including *Acinetobacter* spp., *Aeromonas* spp., *Citrobacter* spp., *Enterobacter* spp., *Escherichia coli*, *Klebsiella* spp., *Kluyvera* spp., *Proteus* spp., *Providencia* spp., *Pseudomonas* spp., *Raoultella* spp., and *Salmonella* spp., isolates [11]. However, some of these *mcr* variants do not cause colistin resistance [12, 13].

The *mcr* genes are harboured by different conjugative and non-conjugative plasmids and have been found to be integrated into the chromosome in some isolates [14]. In addition to clinical isolates, *mcr* genes have been detected in various isolates from environmental sources including water, soil, livestock, vegetables, flies, wildlife, companion animals and birds [1, 2, 15–24].

The *mcr-1* gene has been identified in clinical isolates in multiple hospitals across South Africa, [19, 25, 26] however data regarding the distribution of the *mcr* genes in the environment in South Africa is limited. *mcr-1* has been detected in *E. coli* isolated from broiler chickens, a pig and final effluents from wastewater treatment plants in South Africa [27–30]. The presence of *mcr* genes in the environment could be a reservoir for colistin resistance in clinical settings.

This study investigated the presence of colistin resistant GNB and *mcr* genes in samples from three surface water sources, the Plankenburg-, Eerste- and Berg rivers, as well as storm water from Muizenberg and Fish Hoek in the Western Cape province, South Africa, and characterised the role of the *mcr* variants in colistin resistance. Informal housing schemes are situated on the banks of these rivers, which are highly polluted by sewage, industrial and agricultural run-off [31], while storm water systems are often used for the illegal disposal of human waste. As such, the presence of colistin resistance mechanisms in these water sources provides insight into the dissemination of colistin resistance in communities and poses a risk for further dissemination of resistance genes.

## Methods

### Isolate collection

**Fig. 1:**
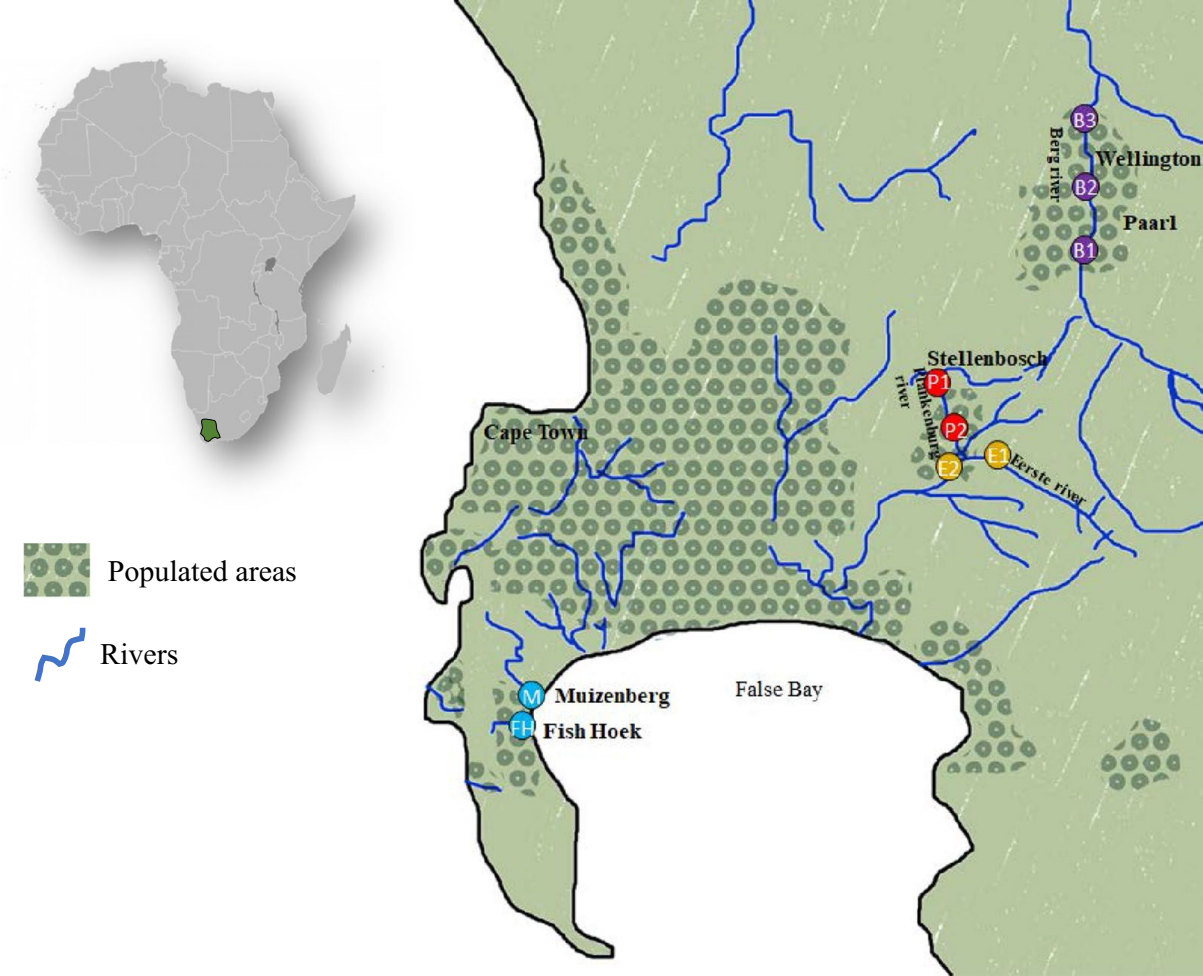
Overview of the sampling region in the Western Cape of South Africa. Sampling sites were situated in the Plankenburg river (P1-before a community and P2-after a community) and Eerste river (E1-before a community and E2-after a community) in Stellenbosch and three sampling sites (B1–B3) from three communities from the Berg river in Paarl and Wellington and two storm water sampling sites (M and FH) in Muizenberg and Fish Hoek respectively

The Plankenburg, Eerste and Berg rivers and tributaries were sampled between May 2019 and January 2020 (Fig. 1). Sample sites P1 in the Plankenburg river and E1 in the Eerste river are situated above two separate Stellenbosch communities, while sample sites P2 and E2 are situated below the respective communities. Upstream and downstream community samples were sampled on the same day, approximately 1 h apart. The samples were taken at 30 cm depth, except if the water depth was less than 30 cm, the samples were taken at the halfway point between the water surface and the bottom of the water body. Special care was taken to open the sample bottle only at the correct sample depth to avoid capturing floating contamination. Rivers were only sampled once, except the Plankenburg river which was sampled twice, in May 2019 (P2a) and January 2020 (P2b). No upstream sample was obtained in January 2020 as the river was dry upstream of the community. We thus also used the 2019 samples in this study, as they had been collected both upstream and downstream. Sampling sites B1, B2 and B3 in the Berg river are situated in three different communities: Paarl (Boulevard), Paarl (Mbekweni) and Wellington, respectively. Storm water from Muizenberg (M) and Fish Hoek (FH) was sampled in November 2019 (Fig. 1 and Additional file 1: Table 1). Sampling sites were chosen based on representativity and accessibility. Sampling took place during dry weather conditions in order to get an accurate representation of the water in an undiluted state. The water temperature, turbidity, river flow speed and other visible problems were documented in conjunction with recent weather patterns and are included in Additional file 1: Table 1. Water was collected in sterile bottles, kept on ice during transport and subsequently refrigerated at 4 °C.

### Bacterial enumeration and culture-based screening

*E. coli* and total coliform counts were performed on the water samples using the Colilert-18 water test as per manufacturer’s instructions (IDEXX, South Africa). Colilert-18 is a semi-automated quantitative method which uses the metabolism of nutrient indicators o-nitrophenyl (ONPG) and 4-methyl-umbelliferyl (MUG) to detect coliforms and *E. coli*, respectively, using colorimetric changes and fluorescence. The South African national guidelines for water quality guidelines for full recreational contact, indicates a threshold of > 130 cfu/100 ml of definite bacterial growth in the water system as a significant and increased risk of infectious disease transmission [32].

Serial dilutions (10^−1^–10^−6^) of each water sample were plated out (0.1 ml) in triplicate on MacConkey agar (MCC, Sigma-Aldrich, South Africa) and MacConkey agar containing 10 mg/L vancomycin and 2 mg/L colistin (Mac-Col2-Van10, Sigma-Aldrich), using the spread plate method. Vancomycin was included to eliminate the growth of most Gram-positive bacteria and colistin was used to select for colistin resistant organisms. Plates were incubated aerobically at 37 °C for 18–22 h. Colonies were also enumerated on the MCC plates in order to compare the growth on plates with and without antibiotics and to determine the proportion of colistin resistant GNB isolates in the water.

### Selection of colistin resistant bacteria

Distinct bacterial colonies were selected based on their morphological appearance on Mac-Col2-Van10 agar. The colonies were inoculated onto UriSelect agar (NHLS Media Laboratory, Green Point, South Africa) for preliminary identification after incubation aerobically at 37 °C for 18–22 h. Colonies were excluded if they were any colour other than pink (indicative of *E. coli*) or blue (indicative of *Klebsiella/Enterobacter/Serratia/Citrobacter* spp.) on the UriSelect agar plate, e.g. white, cream or brown. Matrix assisted laser desorption ionization-time of flight (MALDI-TOF, Bruker) was performed to confirm the identification of all isolates. Intrinsically colistin resistant organisms, specifically *Serratia* spp. and *Providencia rettgerii,* were excluded from further analysis.

### Susceptibility testing

Colistin resistance was confirmed by broth microdilution (BMD) following the European Committee on Antimicrobial Susceptibility Testing (EUCAST) guidelines and breakpoints version 10 (ISO-standard broth microdilution method 20776-1) [33]. Isolates with a minimum inhibitory concentration (MIC) of ≤ 2 mg/L were classified as colistin susceptible and > 2 mg/L as colistin resistant. *E. coli* ATCC® 25922 and NCTC® 13846 were used as controls.

### Molecular detection of the *mcr* genes

DNA was extracted directly from each water sample using the DNeasy PowerWater Kit (Qiagen, South Africa) on the day of sampling and from the isolates using a crude DNA extraction method (boil-freeze) [34].

Multiplex PCR detection of the *mcr-1, -2, -3, -4* and *-5* genes was performed on all isolates, as well as directly on the water samples, using previously described protocols and controls [35] (Table 1). Singleplex PCR detection of the *mcr-6, -7* and *-8* genes was performed using previously designed primers (Table 1) and *mcr-9* was detected using MCR-9YF and MCR-9YR (Table 1), however no positive controls were available for these genes. Sanger sequencing was performed on all *mcr* amplicons to determine the *mcr* variants present.

**Table 1:** Primers used in this study

Target gene	Primer name	DNA sequence (5′ → 3′)	Amplicon size (bp)	Reference
*mcr-1*	CLR5-F	5′-CGGTCAGTCCGTTTGTTC-3′	309	[1]
	CLR5-R	5′-CTTGGTCGGTCTGTAGGG-3′		
	MCR-1YF	5′-CATATGATGATGCAGCATACTTCT GTGTGGTACCG-3′	1632	This study
	MCR-1YR	5′-CTCGAGTCAGCGGATGAATGCGGT-3′		
*mcr-2*	MCR2-IF	5′-TGTTGCTTGTGCCGATTGGA-3′	567	[2]
	MCR2-IR	5′-AGATGGTATTGTTGGTTGCTG-3′		
*mcr-3*	MCR3-F	5′-TTGGCACTGTATTTTGCATTT-3′	542	[3]
	MCR3-R	5′-TTAACGAAATTGGCTGGAACA-3′		
	MCR-3YF	5′-ATGCCTTCCCTTATAAAAATAAA-3′	1622	This study
	MCR3-YR2	5′-TCAATTATTCCGACATTGCTTA-3′		
	MCR-3YF2	5′-TGAAAGGGGATAAGCTGGTT-3′	Not applicable	This study
	MCR-3YR3	5′-CATAATCTTGATAGTATAGTGCTG-3′		
	MCR-3 YF3	5′-CATATGATGCCTTCCCTTATAAAA ATAAAAATTGT-3′	1622	This study
	MCR-3 YR4	5′-CTCGAGTCAATTATTCCGACATTGC-3′		
*mcr-4*	MCR-4 FW	5′-ATTGGGATAGTCGCCTTTTT-3′	487	[36]
	MCR-4 RV	5′-TTACAGCCAGAATCATTATCA-3′		
*mcr-5*	MCR5_FW	5′-ATGCGGTTGTCTGCATTTATC-3′	1644	[5]
	MCR5_REV	5′-TCATTGTGGTTGTCCTTTTCTG-3′		
*mcr-6*	MCR-6F	5′-GTCCGGTCAATCCCTATCTGT-3′	556	[8]
	MCR-6R	5′-ATCACGGGATTGACATAGCTAC-3′		
*mcr-7*	MCR-7F	5′-TGCTCAAGCCCTTCTTTTCGT-3′	892	
	MCR-7R	5′-TTCATCTGCGCCACCTCGT-3′		
*mcr-8*	MCR-8F	5′-AACCGCCAGAGCACAGAATT-3′	667	
	MCR-8R	5′-TTCCCCCAGCGATTCTCCAT-3′		
*mcr-9*	MCR-9YF	5′-ATGCCTGTACTTTTCAGGGTGAAAG-3′	1619	This study
	MCR-9YR	5′-TTCCGCGAATGCCGTGGCTAA-3′		

The entire *mcr-3* and *mcr-5* genes were amplified and sequenced using primers MCR-3YF and MCR3-YR2, and previously described *mcr-5* gene primers MCR5_FW and MCR5_REV [5], respectively (Table 1). The PCR reactions were performed using the KAPA2G Fast Multiplex PCR Kit (Kapa Biosystems) with 0.4 μM of each primer in a 25 μL reaction volume, with an annealing temperature of 60 °C for 30 cycles. Amplicons were separated on a 1.5% w/v agarose gel and detected using the Alliance 2.7 imaging system (UVITec). Additional internal primers (MCR-3YF2 and MCR-3YR3, Table 1) were designed to complete the sequencing of the *mcr-3* gene.

### *mcr-3* variant functionality assay

Novel *mcr-3* gene variants with unknown colistin susceptibility functionality were cloned and expressed to determine their functionality. The entire 1623 bp *mcr-3* coding sequence was amplified using primers MCR-3 YF3 and MCR-3 YR4 (IDT, USA, Table 1) using the HIFI HotStart ReadyMixPCR kit (Kapa Biosystems, South Africa) with 0.2 μM of each primer and an annealing temperature of 60ºC. The *mcr-1* gene was amplified from NCTC® 13846 as a control using primers MCR-1YF, MCR-1YR (Table 1) with the same PCR conditions and an annealing temperature of 58 °C.

The *mcr* genes were cloned into the XhoI and NdeI restriction sites of the pET-48b(+) expression vector via the CloneJET PCR Cloning Kit (Thermo Fisher Scientific, Difco Laboratories, US) [37] and transformed into a colistin susceptible SHuffle T7 Competent *E. coli* strain (Inqaba Biotechnical Industries (Pty) Ltd, South Africa). Colonies were selected on 15 mg/L kanamycin Luria–Bertani (LB) agar plates and the presence of the *mcr* gene was confirmed using PCR. Colistin susceptibility of the recombinant *E. coli* was determined in triplicate, using BMD.

## Results

The river and storm water samples were heavily contaminated with bacteria, as they contained an abundance of *E. coli* and coliforms (Additional file 1: Table 2). The total *E. coli* count from all water sources ranged between 50 cfu/100 mL–450000 cfu/100 mL and the total coliform counts ranged between 550 cfu/100 mL–2400000 cfu/100 mL. These counts considerably exceeded the South African guidelines of < 130 cfu/100 ml for full recreational contact. The coliform count was higher in the water samples collected downstream as compared to samples taken upstream of communities (~ 1000-fold in the Plankenburg river, 42-fold in the Eerste river). Water samples from the Berg river and storm water samples from Muizenberg and Fish Hoek, in which sampling sites were situated alongside communities, also contained high coliform counts (2600–2,400,000 cfu/mL, 45,000 cfu/mL and 12,700 cfu/mL, respectively) (Additional file 1: Table 2). Two sites closest to the informal settlements of Kayamandi in Stellenbosch (Plankenburg River, site P2) and Mbekweni in Paarl (Berg river, site B2) contained the most coliforms and *E. coli,* based on the Colilert 18® results (Fig. 1 and Additional file 1: Table 2). The proportion of colistin resistant colonies based on the colony counts on MCC vs. Mac-Col2-Van10 (Additional file 1: Table 2), ranged between 5–80% and are given in Table 2.

**Table 2:** Colistin resistant isolates and *mcr-3* and *mcr-5.1* colistin resistance genes detected in river and storm water in the Western Cape of South Africa during 2019–2020

Sampling sites	Plankenburg river			Eerste river		Berg river			Muizenberg storm water	Fish Hoek storm water
	P1	P2a	P2b	E1	E2	B1	B2	B3	M	FH
Sampling date	May 2019	May 2019	Jan 2020	Jan 2020	Jan 2020	Jan 2020	Jan 2020	Jan 2020	Nov 2019	Nov 2019
Proportion of phenotypically colistin resistant colonies, n (%)	496/620 (80%)	5670/13000 (44%)	7900/42000 (19%)	400/1050 (38%)	85,000/188000 (45%)	840/1350 (62%)	14,700/186000 (7.9%)	540/1480 (36%)	320/6400 (5%)	170/940 (18%)
Colistin resistant isolates (n, MIC)	*Aeromonas jandei* (3, MIC: 32), *Aeromonas sobria* (3, MIC: 32), *Aeromonas hydrophila* (2, MIC: 64), *Pectobacterium carotovorum* (1, MIC: 16)	*Raoultella ornithinolytica* (10, MIC: 64), *Franconibacter helveticus* (2, MIC: 64), *Kluyvera ascorbata* (2, MIC:16), *Citrobacter freundi* (1, MIC:16), *Klebsiella oxytoca* (1, MIC:16), *Pantoea agglomerans* (1, MIC:16), *Pluralibacter gergoviae* (1, MIC:16))	*Aeromonas hydrophila* (11, MIC: 64), *Salmonella enterica* (1, MIC: 64)	*Aeromonas hydrophila* (2, MIC: 64)	*Aeromonas hydrophila* (11, MIC: 32 or 64), *Klebsiella oxytoca* (1, MIC:4)	*Aeromonas jandei* (5, MIC: 64), *Raoultella ornithinolytica* (4, MIC: 4 or 64), *Aeromonas hydrophila* (3, MIC: 32 or 64), *Klebsiella oxytoca* (1, MIC: 8), *Pectobacterium carotovorum* (1, MIC: 16)	*Aeromonas hydrophila* (3, MIC: 64)	*Aeromonas hydrophila* (5, MIC: 64), *Aeromonas veronii* (4, MIC: 4 or 8), *Aeromonas sobria* (3, MIC: 4), *Aeromonas jandei* (1, MIC: 4)	*Aeromonas hydrophila* (13, MIC: 32 or 64)	*Aeromonas hydrophila* (1, MIC: 64), *Enterobacter kobei* (1, MIC: 64)
Number of isolates of each species in which *mcr* genes were detected (n)	None detected	None detected	None detected	None detected	None detected	*A. jandei* *mcr-3.33* (1) *mcr-3.34* (1) *mcr-3.35* (1) *mcr-3.36* (2)	None detected	*A. veronii* *mcr-3.37* (1)	None detected	None detected
*mcr* genes detected directly from the water sample (eDNA)	None detected	*mcr-3* *mcr-5.1*	*mcr-3* *mcr-5.1*	None detected	*mcr-3* *mcr-5.1*	*mcr-3*	None detected	*mcr-3* *mcr-5.1*	*mcr-3* *mcr-5.1*	*mcr-5.1*

Following exclusion of intrinsically colistin resistant bacteria and BMD confirmation of colistin resistance, a total of 98 isolates were screened for *mcr* genes, including 39 from the Plankenburg river, 14 from the Eerste river, 30 from the Berg river, 13 from Muizenberg storm water and two from Fish Hoek storm water (Table 1 and Additional file 1: Table 2). *Aeromonas* spp. (70/98, 71%) was the most common colistin resistant GNB isolated from the river and storm water samples (Table 2). No colistin resistant *E. coli* isolates were detected and only two colistin resistant *Klebsiella oxytoca* isolates were isolated from river water at the P2a and E2 sites.

*mcr-3* was detected in six isolates, including five *Aeromonas jandei* isolates (BB, BL, BT, BCC and BMM) and one *Aeromonas veronii* isolate (S2K), all from the Berg river samples (Table 2). The Sanger sequences of the *mcr-3* genes were uploaded to GenBank as new allele variants and allele curation was requested. Five novel *mcr-3* gene variants were detected; *mcr-3.33* (BB: MT791039, n = 1), *mcr-3.34* (BL: MT791040, n = 1), *mcr-3.35* (BT: MT809044, n = 1), *mcr-3.36* (BCC: MT809046 and BMM: MT809045, n = 2) and *mcr-3.37* (S2K: MT809047, n = 1) (Table 2). Recombinant expression of *mcr-3.33, mcr-3.34, mcr-3.35, mcr-3.36* and *mcr-3.37* in *E. coli* showed that all of the variants confer resistance to colistin, resulting in an eightfold increase in colistin MIC (Table 3). No *mcr* genes were detected in the remaining colistin resistant isolates.

**Table 3:** The effect of recombinant *mcr-3* variant expression on colistin MIC, based on BMD

Isolates	Colistin BMD MIC (mg/L)
*E. coli* ATCC 25922 (susceptible control)	0.5
*E. coli* NCTC® 13846 (*mcr-1* control)	4
*E. coli* SHuffle T7	0.5
*E. coli SHuffle T7 transformed with:*	
pET-48b(+)	0.5
pET-48b(+)-*mcr-1*	4
pET-48b(+)-*mcr-3.33*	4
pET-48b(+)-*mcr-3.34*	4
pET-48b(+)-*mcr-3.35*	4
pET-48b(+)-*mcr-3.36*	4
pET-48b(+)-*mcr-3.37*	4

MIC ≤ 2 mg/L: susceptible, MIC > 2 mg/L: resistant

Based on the DNA extracted directly from the water, the *mcr-3* and *mcr-5.1* genes were detected in the Plankenburg river (sites P2a and P2b), the Eerste river (site E2) and the Berg river (site B3), while another site in the Berg river (site B1) contained only *mcr-3*. The *mcr* genes were only detected in river water samples taken downstream of, or alongside communities, and not upstream of the communities. The *mcr-5.1* gene was detected in both storm water samples from Muizenberg and Fish Hoek and the Muizenberg storm water sample also contained *mcr-3* (Table 2). All of the *mcr-5* genes shared 100% identity with the *mcr-5.1* variant found in *Salmonella enterica* subsp. *enterica* serovar Paratyphi B (GenBank: KY807921).

## Discussion

The river and storm water investigated in this study were heavily contaminated with faecal bacteria as evidenced by elevated *E. coli* and coliform levels (Additional file 1: Table 2). *E. coli* counts are a definitive indication of faecal contamination. High counts pose a public health risk and are associated with transmission of infectious diseases. Many South African rivers have been found to be unsuitable as a raw source for purification for drinking water, or for irrigation and other recreational purposes [31] and our findings support previous reports on the high level of bacterial contamination in water sources [31, 38].

Comparison of the samples collected up- and downstream of communities on the Plankenburg and Eerste rivers, provides an indication of the contamination introduced by runoff from the communities, and can be used as an indication of the carriage of resistance in the communities. The coliform and *E. coli* enumerations showed that there was substantial faecal contamination of the water between the two collection sites as well as in the Berg river and Fish Hoek and Muizenberg storm water systems (Additional file 1: Table 2). These water sources are highly polluted with various genera of bacteria, especially where these water sources are used for irrigational and/or recreational purposes (sites P2, E2, B2 and B3). This poses a significant public health risk via numerous pathways: direct exposure, irrigation of edible food crops, livestock watering and exposure to raw water usage by industry.

*Aeromonas spp.* was the most prevalent colistin resistant organism detected in this study. *Aeromonas hydrophila* plays a key role in degrading carcinogenic, teratogenic and mutagenic polycyclic aromatic hydrocarbons (PAHs) in water, especially acenaphthene and fluorine which are used to bioremediate PAH contaminated river systems [39]. However, *Aeromonas* spp. are opportunistic pathogens and have been found to be responsible for a variety of infectious complications such as gastroenteritis, sepsis, meningitis, respiratory and genitourinary infections, wound infections and infections of skin and soft tissue [40].

*mcr-3* and *mcr-5.1* gene variants were found to be widely dispersed in water sources in the study area, based on direct screening for these genes in the water samples. These genes were detected in water which is contaminated by communities, and not at upstream sites from communities. Although, the prevalence of *mcr* genes in the colistin resistant isolates was low, novel *mcr-3* variants conferring colistin resistance were detected in 6 *Aeromonas spp.* isolates from the Berg river. The *mcr-3* gene has been previously detected in *Aeromonas spp*. from fish and turkey in 2005, 2006, 2008 and 2012 in Germany [41] and from humans, retail meat, and environmental water samples in 2016 and 2017 in China [14, 42–44]. Two *mcr-5* positive *A. hydrophila* were previously isolated from a pig faecal sample and hospital sewage in China in 2017 and 2014, respectively [45, 46]. No *mcr-1-5* genes were detected in any of the other 92 colistin resistant isolates, suggesting that the mechanism of colistin resistance could be chromosomal in nature or conferred by additional recently (*mcr-10*) or undescribed *mcr* genes. However, there is limited knowledge on the mechanisms of colistin resistance in the genera of bacteria isolated in this study. The *mcr* genes could also be present in bacteria that are viable but non culturable (VBNC) or in the isolates that were excluded during the selection process and therefore can only be found when performing PCR screening directly on the water samples. Intrinsically colistin resistant organisms have shown to be potential traffickers of *mcr* genes [21, 47]. In our study we excluded a *Providencia rettgerii* (n = 1) and *Serratia* spp. (n = 8) (Additional file 1: Table 2), however these intrinsically colistin resistant could be carrying *mcr* genes and should be further investigated in the future.

The *mcr-3* and *mcr-5* genes described in this study have not previously been described in any sample type in South Africa, however, this study suggests that the *mcr-3* and *mcr-5.1* genes are commonly carried in the environment. Only *mcr-1* genes have been described in clinical *E. coli* and *Klebsiella* spp isolates [19, 25], in contrast with these findings. This suggests that the environment carries different colistin resistance genes to those present in hospitalised patients. However, patients could present with infections associated with these unique colistin resistance genes in the near future. Active surveillance is therefore recommended to determine the prevalence of *mcr* genes in clinical isolates.

The plasmid transmissibility of the *mcr* genes means they can be easily transported to a variety of organisms, including those that are clinically significant. Although none of the *E. coli* were colistin resistant, they can possibly become colistin resistant via horizontal gene transmission of the *mcr* genes. The presence of colistin resistant isolates and genes conferring this resistance in the water may be linked to high-density industrial farming practises, involving routine use of antibiotics therapeutically, prophylactically and for growth promotion.

In many areas of South Africa, and worldwide, informal housing schemes are often established along the banks of river systems. Due to a lack of adequate sanitation and waste removal facilities in the informal settlements, as well as poor management and disposal of sewage, the storm water drainage pipes leading directly to the rivers are often used as a means of disposal of human and animal waste. Contamination of colistin resistant bacteria in river and storm water catchments is a cause of great concern, especially to surrounding communities, farms and industries, as in South Africa, river water is used for irrigation and domestic purposes, often without treatment. Controlling and preventing the spread of antimicrobial resistance requires a holistic approach, inclusive of environmental reservoirs of resistant organisms.

## Conclusions

Several colistin resistant GNB, mainly *Aeromonas* spp. isolates, were isolated from three different rivers and two storm water samples in the Western Cape province of South Africa, suggesting that environmental water sources could serve as a reservoir and/or distribution network for colistin resistance. Mobile colistin resistance genes (*mcr-3* and *mcr-5*) in opportunistically pathogenic bacteria (e.g. *Aeromonas* spp.) from these water sources can be horizontally transferred to other human pathogens which could present a potential human health risk. The high levels of colistin resistance in the *mcr* negative bacterial isolates suggests that there are alternative mechanisms of colistin resistance in these environmental bacteria. The presence of colistin resistance and mobile colistin resistance genes in these water sources raises major health concerns as human population densities along these water sources are high, thus resulting in increased human exposure to these organisms which are resistant to a last-resort antibiotic. The need to strengthen and monitor water treatment systems, proper sanitation and waste removal in order to improve the health of the communities living along various water sources is well understood. Our findings highlight the potential role of contaminated water sources in the transmission of resistant organisms. Improving water quality has the potential to both reduce infections in the community as well as to reduce the transmission of resistant organisms.

## Supplementary Information

**Additional file 1**. **Supplementary Table 1.** Field notes on water samples. **Supplementary Table 2.** Colistin resistant isolates collected in river and storm water in the Western Cape of South Africa during 2019–2020.

## Data Availability

All data generated or analysed during this study are included in this published article and its supplementary information files.

## Abbreviations

BMD: Broth microdilution
cfu: Colony forming unit
EUCAST: European Committee on Antimicrobial Susceptibility Testing
GNB: Gram-negative bacteria
LB: Luria–Bertani (LB)
MALDI-TOF: Matrix assisted laser desorption ionization-time of flight
Mac-Col2-Van10: MacConkey agar containing 10 mg/L vancomycin and 2 mg/L colistin
MCC: MacConkey agar
*mcr*: Mobile colistin resistance
VBNC: Viable but non culturable
