# Supplementary material for: Characterisation of mobile colistin resistance genes (mcr-3 and mcr-5) in river and storm water in regions of the Western Cape of South Africa
Source: Antimicrob Resist Infect Control. 2021 Jun 29;10:96. doi: 10.1186/s13756-021-00963-2 (PMC8244157; doi:10.1186/s13756-021-00963-2)
Supplement: Supplementary file 1 — Additional file 1. Supplementary Table 1. Field notes on water samples. Supplementary Table 2. Colistin resistant isolates collected in river and storm water in the Western Cape of South Africa during 2019–2020. [file 13756_2021_963_MOESM1_ESM.docx]

**Supplementary Table 1.** Field notes on water samples

| **Sampling site** | **Date** | **Water temp** | **Turbidity (observed)** | **Flow speed**  **(observed)** | **Remarks - note possible upstream pollution sources and other visible problems** | **Recent weather patterns** |
| --- | --- | --- | --- | --- | --- | --- |
| **P1**, Plankenburg river- below Nooitgedacht Farm | 29 May 2019 | 9˚C | Turbid (brown) | Stagnant | Above urban development. Lower pollution expected | Autumn. Dry conditions, except for one day of good precipitation. Last rained on 19 May (± 40mm). Night minimums 8˚C -19.9˚C over the past week. Day maximums from 19˚C to 27˚C. Winter has not formally set in yet. |
| **P2**, Plankenburg river- Adam Tas Road, above Gilbeys | 29 May 2019 | 10.5˚C | Slightly turbid (grey) | Flowing | Expected severe pollution (faecal + unknown) |  |
|  | 20 Jan 2020 | 15.5˚C | Slightly turbid (grey) | Flowing | Expected severe pollution (faecal + unknown) | Mid-summer. Very high temperatures over the previous few days (>35˚C). Very dry conditions for the past month. Severe windstorm just before sampling. Rained 3mm day before sampling. Night minimums 14˚C -22˚C over the past week. Day maximums 19.2˚C -40.7˚C. |
| **E1**, Eerste river Koloniesland | 20 Jan 2020 | 15˚C | Clear | Medium | - |  |
| **E2**, Eerste river after merge | 20 Jan 2020 | 16˚C | Fairly turbid | Medium | - |  |
| **B1**, Berg River Boulevard | 30 Jan 2020 | 19˚C | Clear | Medium | Bad smell. | Mid-summer. Usual summer temperatures over the previous few days (±30˚C). Dry conditions for the past month except for 3mm of rain 5 days before collection. Night minimums, 13˚C -19˚C, over the past week. Day maximums from 24˚C to 33˚C. Water from the Berg River Dam is released into the river from time to time to improve the water levels for irrigation. This may temporarily affect the pollution levels. |
| **B2**, Tributary Berg River near Paarl Sewage Works | 30 Jan 2020 | 13.5˚C | Fairly turbid | Medium | Bad smell, slime layer on bottom. |  |
| **B3**, Tributary Berg River Wellington | 30 Jan 2020 | 17˚C | Slightly turbid | Slow | - |  |
| **M**, Muizenberg | 21 Nov 2019 | 17˚C | Clear | Slow | Draining from foothills and a few street blocks of housing. | Early summer conditions. No rain the previous 2 weeks. Recent minimums low for this time of the year (10.3˚C the previous day). Day maximums in middle to high twenties (28˚C the previous day). |
| **FH**, Fish Hoek | 21 Nov 2019 | 18˚C | Dirty, anaerobic | Stagnant | On edge of parking lot, sewage contamination. |  |

**Supplementary Table 2.** Colistin resistant isolates collected in river and storm water in the Western Cape of South Africa during 2019-2020.

|  | **Plankenburg river** | | | **Eerste river** | | **Berg river** | | | **Muizenberg storm water** | **Fish Hoek storm water** |
| --- | --- | --- | --- | --- | --- | --- | --- | --- | --- | --- |
| Sites | P1 | P2a | P2b | E1 | E2 | B1 | B2 | B3 | M | FH |
| Sampling date | May 2019 | May 2019 | Jan 2020 | Jan 2020 | Jan 2020 | Jan 2020 | Jan 2020 | Jan 2020 | Nov 2019 | Nov 2019 |
| Total *E. coli* count (cfu/100 mL) | 50 | 26500 | 450000 | 700 | 19000 | 100 | 300000 | 1300 | 9000 | 540 |
| Total coliform count (cfu/100 mL) | 550 | 490000 | 690000 | 1000 | 42000 | 12000 | 2400000 | 2600 | 45000 | 12700 |
| Colony counts on MCC (cfu/100 mL) | 620 | 13000 | 42000 | 1050 | 188000 | 1350 | 186000 | 1480 | 6400 | 940 |
| Colony counts on Mac-Col2-Van10 (cfu/100 mL) | 496 | 5670 | 7900 | 400 | 85000 | 840 | 14700 | 540 | 320 | 170 |
| Isolates picked on Mac-Col2-Van10 (n) | 30 | 76 | 54 | 12 | 28 | 35 | 34 | 36 | 24 | 6 |
| Putative *E. coli* or *Klebsiella* spp. on UriSelect agar (n) | 9 | 18 | 16 | 5 | 14 | 16 | 10 | 21 | 13 | 2 |
| Intrinsically colistin resistant (MALDI-TOF) | None | None | 1 *Providencia rettgerii*  2 *Serratia macescens* | None | 1 *Serratia macescens* | 2 *Serratia* spp. | 2 *Serratia* spp. | 1 *Serratia* spp. | None | None |
| Colistin susceptible isolates (BMD) | None | None | 1 *Comamonas testosterone* | 3 *Aeromonas* spp. | 1 *Raoultella ornitica* | None | *5 Aeromonas* spp. | *7 Aeromonas* spp. | None | None |
| Colistin resistant isolates in this study (n) | 9 | 18 | 12 | 2 | 12 | 14 | 3 | 13 | 13 | 2 |

cfu: colony-forming unit, MCC: MacConkey agar, Mac-Col2-Van10: MacConkey agar with 2 mg/L colistin and 10mg/L vancomycin, BMD: Broth microdilution.
